# The Pestivirus RNase E^rns^ Tames the Interferon Response of the Respiratory Epithelium

**DOI:** 10.3390/v16121908

**Published:** 2024-12-11

**Authors:** Guillaume Beilleau, Hanspeter Stalder, Lea Almeida, Blandina I. Oliveira Esteves, Marco P. Alves, Matthias Schweizer

**Affiliations:** 1 Institute of Virology and Immunology, Länggass-Str. 122, CH-3001 Bern, Switzerland; 2 Department of Infectious Diseases and Pathobiology, Vetsuisse Faculty, University of Bern, CH-3001 Bern, Switzerland; 3 Graduate School for Cellular and Biomedical Sciences, University of Bern, CH-3012 Bern, Switzerland; 4 Multidisciplinary Center for Infectious Diseases, University of Bern, CH-3012 Bern, Switzerland

**Keywords:** pestivirus, bovine viral diarrhea virus (BVDV), viral endonuclease, innate immune evasion, interferon type-I, IFN antagonist, glycosaminoglycan (GAG)-binding site, viral RNase, respiratory epithelium, air–liquid interface (ALI)

## Abstract

Bovine viral diarrhea virus (BVDV), a pestivirus in the family *Flaviviridae*, is a major livestock pathogen. Horizontal transmission leads to acute transient infections via the oronasal route, whereas vertical transmission might lead to the birth of immunotolerant, persistently infected animals. In both cases, BVDV exerts an immunosuppressive effect, predisposing infected animals to secondary infections. E^rns^, an immunomodulatory viral protein, is present on the envelope of the virus and is released as a soluble protein. In this form, it is taken up by cells and, with its RNase activity, degrades single- and double-stranded (ds) RNA, thus preventing activation of the host’s interferon system. Here, we show that E^rns^ of the pestiviruses BVDV and Bungowannah virus effectively inhibit dsRNA-induced IFN synthesis in well-differentiated airway epithelial cells cultured at the air–liquid interface. This activity was observed independently of the side of entry, apical or basolateral, of the pseudostratified, polarized cell layer. Virus infection was successful from both surfaces but was inefficient, requiring several days of incubation. Virus release was almost exclusively restricted to the apical side. This confirms that primary, well-differentiated respiratory epithelial cells cultured at the air–liquid interface are an appropriate model to study viral infection and innate immunotolerance in the bovine respiratory tract. Furthermore, evidence is presented that E^rns^ might contribute to the immunosuppressive effect observed after BVDV infections, especially in persistently infected animals.

## 1. Introduction

Pestiviruses are a group of viruses belonging to the family *Flaviviridae*, which also includes important human pathogens such as hepatitis C virus and dengue virus. Originally, the genus *Pestivirus* was composed of four species, BVDV-1, BVDV-2, classical swine fever virus (CSFV), and border disease virus (BDV). However, the identification of additional species has prompted a reclassification of the genus, first with letters to reflect the varying host tropism (e.g., Pestivirus A for BVDV-1) [1], and more recently, to the ICTV-mandated binomial [2]. The original four species, BVDV-1, BVDV-2, CSFV, and BDV, are now classified as *Pestivirus bovis* (formerly *Pestivirus A*), *Pestivirus tauri* (formerly *Pestivirus B*), *Pestivirus suis* (formerly *Pestivirus C*), and *Pestivirus ovis* (formerly *Pestivirus D*), respectively. Some of the newly identified pestivirus species are now named as *Pestivirus antilocaprae* (pronghorn antelope pestivirus, formerly *Pestivirus E*), *Pestivirus australiaense* (Bungowannah virus, formerly *Pestivirus F*), *Pestivirus brazilense* (HoBi-like pestivirus, formerly *Pestivirus H*), or *Pestivirus scrofae* (atypical porcine pestivirus, formerly *Pestivirus K*), to name just a few.

Among the pestiviruses, BVDV, first described in 1946 [3,4], stands out as a significant pathogen in global cattle populations, causing diverse clinical manifestations such as diarrhea, respiratory symptoms, and reproductive disorders [5,6,7]. These symptoms result in substantial economic losses for the livestock industry worldwide, including reduced productivity, higher veterinary costs, and potential trade restrictions [8,9,10]. BVDV can cause both transient (TI) and persistent infections (PI). TIs typically occur through the oronasal route, while PIs result from vertical transmission from the dam to the fetus, with affected PI animals born serving as long-term reservoirs [11,12,13]. Genetic mutations or viral recombination in PI-infected animals can lead to a cytopathic biotype, which causes the severe and fatal mucosal disease [14,15,16].

Pestiviruses are enveloped viruses whose genome consists of a single-stranded (ss), positive-sense RNA encoding a single open reading frame comprising four structural proteins (C, E^rns^, E1 and E2) and at least eight non-structural (NS) proteins (N^pro^, p7, NS2, NS3, NS4A, NS4B, NS5A, and NS5B) [17]. Pestiviruses replicate using a semi-conservative mechanism with a double-stranded RNA (dsRNA) template to produce plus-strand genomic RNA. This process generates dsRNA intermediates in the cytosol of infected cells [18]. These dsRNA intermediates, along with highly structured viral genomic ssRNA or fragments thereof from decayed virus particles, can act as pathogen-associated molecular patterns (PAMPs) that might stimulate the host’s pattern recognition receptors, including TLR-3, TLR-7/8, and RIG-I-like receptors (RLRs) to activate the host interferon (IFN) response [19,20,21]. The virus evades the host’s innate immune defence by expressing two IFN antagonists: the non-structural protein N^pro^ and the structural protein E^rns^ [22], the latter being the focus of this study. E^rns^ is a highly glycosylated protein with a length of around 225 amino acids and an apparent molecular mass of a monomer of approx. 40–50 kDa, but it usually appears as disulfide-linked homodimers [17]. In addition to its role as envelope glycoprotein, it is an endoribonuclease released in the form of an extracellular soluble protein [23,24]. It belongs to the RNase T2 family of endonucleases and is active in a broad pH range from about 4–7.5 with an optimum around a pH value of 6 ([17,25], and references therein). In virus-infected cells, the non-structural protein N^pro^ inhibits IFN induction by leading to the degradation of the transcription factor IRF-3 [26]. In addition, the soluble protein E^rns^ binds to the cell surface of infected and uninfected cells via its C-terminal GAG-binding domain, is internalized by clathrin-mediated endocytosis and degrades ss- and dsRNA via its endonuclease activity before it can engage with TLR-3 and/or TLR-7/8 in endolysosomal compartments [27,28,29,30].

Horizontal transmission of BVDV mainly occurs through the oronasal route, with the respiratory tract representing an important point of entry that is regularly exposed to microbes, allergens, and particles during inhalation [31]. Infections with BVDV are reported to cause an immunosuppressive effect [32], especially when co-infected with other bacteria or viruses, e.g., for the latter, bovine respiratory syncytial virus, parainfluenza type-3 virus, influenza D virus (IDV), bovine herpesvirus-1, or bovine coronavirus. Thus, BVDV might contribute to the well-known bovine respiratory disease complex (BRDC) leading to severe respiratory diseases [33,34,35]. The molecular mechanisms of immunosuppression that is observed after transient and persistent infection is still a matter of debate but might well include a multitude of factors [36,37]. As soluble E^rns^ can be detected in the oronasal secretions of PI animals [38], it might well be envisaged that this viral RNase plays an important role in the immunosuppressive effect by inhibiting the activation of the innate and, as a consequence, the adaptive immune system. Therefore, our aim was to investigate the activity of E^rns^ as IFN antagonist on differentiated, pseudostratified bovine airway epithelial cells cultured in an air–liquid interface (ALI) system that contains ciliated cells, goblet cells, and basal cells. This model system is considered as highly representative of the native airway epithelium [39]. Here, we show that E^rns^ added to the apical and basolateral side of the ALI culture system dose-dependently inhibited dsRNA-induced IFN expression independent of the side of dsRNA application. By contrast, infection of the differentiated airway epithelial cells by various strains of BVDV could be achieved but was not efficient enough to interfere with the IFN response. Finally, the results confirmed along with previous reports that BVDV is largely released only from the apical compartment independent on the side of infection [40], and that E^rns^ does not require a species-specific receptor for its activity [30,41] as it was also active on human epithelial cells cultured in an ALI system. Thus, this ALI cell culture system represents an excellent model to study the interaction of pestiviruses with the host’s innate immune system and will enable further studies to investigate the oronasal transmission of pestiviruses.

## 2. Materials and Methods

### 2.1. Cell Cultures

#### 2.1.1. Primary Bovine Turbinate (BT) Cells

Primary bovine turbinate (BT) cells were generated at our institute from fetuses collected from a local abattoir and were thoroughly screened to confirm the absence of ruminant pestiviruses by immunoperoxidase staining. The BT cells were grown in medium with 7% FCS (PAA Laboratories; Lucerna-Chem AG, Lucerne, Switzerland) as described [42]. Mycoplasma contamination was excluded using the SouthernBiotech Mycoplasma Detection Kit (BioConcept Ltd., Allschwil, Switzerland) according to manufacturer protocol.

#### 2.1.2. Primary Human Bronchial Airway Epithelial Cells (hBAEC) in Air–Liquid Interface (ALI)

Tracheobronchial cells were extracted from patients over the age of 18 who underwent bronchoscopy or pulmonary resection at the Cantonal Hospital in St. Gallen, Switzerland. The isolation and culturing processes adhered strictly to ethical guidelines as described [43]. These primary human bronchial epithelial cells were free of mycoplasma [44], and were cultured and differentiated into a pseudostratified, ciliated epithelium in an air–liquid interface (ALI) system according to standard procedures [45,46].

However, due to supply difficulties with LHC Basal Medium, component of both, the ‘bronchial epithelial cell serum-free growth medium (BEGM)’ and the ‘air–liquid interface (ALI) medium’, we were forced to switch to new culture media from STEMCELL Technologies SARL (Saint Égrève, France). The PneumaCult™-Ex Plus Medium replaced the BEGM, and the PneumaCult™-ALI Medium replaced the previous ‘ALI medium’. We compared the composition of the different media and confirmed experimentally that the proliferation and differentiation capacity of primary human tracheobronchial epithelial cells remained unchanged.

#### 2.1.3. Culture of Primary Bovine Bronchial Airway Epithelial Cells (bBAEC) in Air–Liquid Interface (ALI)

Tracheobronchial epithelial cells were isolated from postmortem tracheobronchial tissue obtained from cattle (*Bos taurus*) at the Institute of Animal Pathology, University of Bern, Switzerland. The procedures followed local regulations and ethical guidelines. In order to minimize the likelihood of finding respiratory viruses in post-mortem tracheobronchial tissue, samples were obtained during the summer period. To confirm the absence of specific common viruses, such as IDV and BVDV, quantitative reverse transcription polymerase chain reaction (RT-qPCR) and immunostaining analyses were performed and found to be negative. The cells were isolated and cultured as detailed in Gultom et al. [46] into a well-differentiated pseudostratified, ciliated epithelium in an ALI system, and were tested free of mycoplasma [44].

### 2.2. Viral Infection of hBAEC and bBAEC Cultured Under ALI Conditions

Well-differentiated bBAEC and hBAEC cultures were infected at a multiplicity of infection (MOI) of three with either the noncytopathic BVDV-1 (*Pestivirus bovis*) strains Ncp7 [47], NADL [48] (kindly provided by C.M. Rice, Rockefeller University, New York City, NY, USA), Suwa [49], and Pe515 [47], and Bungowannah pestivirus (*Pestivirus australiaense*), the latter kindly provided by M. Beer (FLI Riems, Germany, with the permission of P.D. Kirkland, Elizabeth Macarthur Agricultural Institute, Menangle, New South Wales, Australia). IDV (kindly provided by R. Dijkman, Institute of Infectious Diseases, University of Bern, Switzerland) was used as positive infection control [50]. For this, the cells were incubated for three days after infection, as IDV replication was previously reported to reach maximal replication within 72 h post-infection [44]. Viruses were diluted in Hanks balanced salt solution (HBSS; Gibco, Thermo Fisher Scientific, Reinach, Switzerland), inoculated on the apical and basolateral side, and incubated for 3 h and up to 24 h, respectively, at 37 °C. However, the extension of the incubation time at the basolateral side above 3 h did not provide any difference. On the apical (air-exposed) side, incubation longer than 3 h started to compromise the integrity of the cell layer. Afterwards, the virus inoculum was removed, and the apical or basolateral surface was washed three times with HBSS. The third wash was collected as the 1 h post-infection (hpi) time point. The cells were then maintained in a humidified incubator at 37 °C with 5% CO_2_. The viral progeny was harvested at 24 h intervals. For the apical side, 200 μL of HBSS was incubated on the apical surface for 10 min before collection at each specified time point. The apical washes obtained were then mixed in a 1:1 ratio with virus transport medium [46] and stored at -80 °C until further analysis. The basolateral medium was also collected at each time point and stored at −80 °C, and replaced by fresh ALI medium at 37 °C. A subset of infected bBAEC cultures was collected after 48 h or 10 days post-infection (dpi) for immunofluorescence analysis.

The integrity of the barrier function in bBAEC cultures during pestivirus infections was evaluated by monitoring trans-epithelial electrical resistance (TEER) using an EVOM^2^ Epithelial Voltohmmeter (World Precision Instruments, Friedberg, Germany) [46]. Measurements were obtained at day 0 and day 10 dpi. Data indicated no significant differences between infected and non-infected bBAEC cultures throughout the experiments, suggesting that the barrier function remained unaffected by pestivirus infection.

### 2.3. Quantification of Viral RNA by RT-qPCR

RNA extraction was conducted using the NucleoMag VET Kit (Macherey-Nagel, Oensingen, Switzerland) in conjunction with the KingFisher Flex System (Thermo Fisher Scientific). Following extraction, one-step reverse transcription real-time polymerase chain reaction (RT-qPCR) was performed using the QuantiTect^®^ Probe RT-PCR Kit (Qia gen AG, Hombrechtikon, Switzerland) with primers that are used in our Swiss reference laboratory for ruminant pestiviruses targeting the 5′-untranslated region (5′-UTR) of the viral RNA. Amplification parameters were reverse transcription at 50 °C for 30 min; poly merase activation at 95 °C for 15 min; and 45 cycles of denaturation at 94 °C for 15 s and annealing and extension at 60 °C for 1 min. GAPDH served as an internal control when using cell lysates [47,51] with the same amplification parameters using the primers boGAPDH_2f (5′-GGG TGA TGC TGG TGC TGA GT-3′) and 4R (5′-AAG CAG TTG GTG GTG CAG G-3′) and the probe 3rP (FAM) (5′-TGT GAT GGG CGT GAA CCA CGA GAA GTA-3′). To also include a control for the RNA isolation for each supernatant sample, we used the Sendai virus, which provides a control RNA that is protected from RNase degra dation within the virus particle. A specific amount of Sendai virus, sufficient to produce a cycle threshold (Ct) value of approximately 25, was added to each sample before RNA isolation. This allowed us to assess the correct implementation of both, the RNA isolation and the RT-qPCR reaction, for each individual sample [52]. For better visualization, i.e., in order that an increase in the value also represents an increase in the amount of viral RNA, the Ct value obtained was replaced by a value from the calculation ‘45—(Ct value of the sample) divided by the slope of the standard curve (3.428)’.

### 2.4. Virus Titration

Virus titration, according to World Organisation for Animal Health (WOAH) guide lines [53], was performed as described [42]. Briefly, virus samples were serially diluted seven times in ten-fold increments using MEM supplemented with 2% FCS. This process was performed directly in 96-well plates, with each dilution tested in six wells. In addition, six wells were designated as negative controls, into which only the medium was intro duced without inclusion of virus. Next, a suspension of BT cells (20,000 cells per well in 100 μL of MEM with 2% FCS) was introduced into the wells. The plates were incubated for 4–5 days at 37 °C in a humidified atmosphere with 5% CO_2_ followed by immuno peroxidase staining [42]. Virus titers were determined according to the method of Reed and Munch [54] and reported as tissue culture infectious dose 50 (TCID_50_) per mL.

### 2.5. Immunofluorescence Staining on bBAEC and hBAEC Cultured Under ALI Conditions

Immunofluorescence assays were performed following the method established by Gultom et al. [46], with additions to include specific antibodies for the detection of pestivirus and IDV. For pan-pestivirus detection, a commercially available pool of four mouse-derived monoclonal antibodies targeting a broad spectrum of pestivirus antigens (NS2-3 and E2) was employed (RAE 2020, APHA Scientific, UK). As secondary antibody, Alexa Fluor^®^ 488-conjugated donkey anti-Mouse IgG (H + L) (Jackson ImmunoResearch; Milan Analytica AG, Rheinfelden, Switzerland) was used. Alternatively, an antibody to dsRNA (J2, English & Scientific Consulting Kft., Szirák, Hungary) was used as described [55]. To identify IDV, an unconjugated antibody against the nucleoprotein (NP) of the D/bovine/Oklahoma/660/2013 strain (a custom rabbit polyclonal antibody produced by GenScript, Piscataway, NJ, USA) was applied, followed by Alexa Fluor^®^ 488-conjugated donkey anti-Rabbit IgG (H + L) (Jackson ImmunoResearch) as described [50]. Visualization was performed using an EVOS™-FL Auto 2 imaging system (Invitrogen, Thermo Fisher Scientific) with a 40× air objective. Image processing was carried out with Fiji (ImageJ 1.54f, https://imagej.net/ij/).

### 2.6. Inhibition of dsRNA-Induced IFN Induction by E^rns^

#### 2.6.1. Expression and Purification of E^rns^ Proteins

Strep-tagged E^rns^ proteins were expressed in the supernatant of HEK cells in suspension and purified by Strep-tag affinity column purification as described Lussi et al. [56]. In addition to wild-type (wt) E^rns^ of the BVDV-1 strain Ncp7, an RNase-inactive mutant (E^rns^-H30F) and a mutant lacking the C-terminal amphipathic helix and heparin-binding site (E^rns^-ΔC) was used. The characteristics of these E^rns^ preparations, i.e., its RNase activity and the ability to inhibit dsRNA-induced IFN induction, was controlled by in vitro RNase activity assays and by the analysis of Poly IC-induced Mx expression in BT cells [56,57].

#### 2.6.2. Incubation of bBAEC and hBAEC in the Presence of E^rns^

Well-differentiated bBAEC and hBAEC cultures were exposed to fixed concentrations of E^rns^ (2.5 ng/µL and 5 ng/µL). For apical stimulation, E^rns^ was diluted in 200 µL of prewarmed HBSS and applied to the apical chamber, followed by a 90 min incubation at 37 °C in a humidified incubator with 5% CO_2_. For basolateral stimulation, E^rns^ was diluted in 500 µL of prewarmed ALI medium and introduced to the basolateral chamber, with incubation times ranging from 6 to 24 h under the same conditions. After incubation, the cells were washed once to remove any soluble extracellular proteins. Subsequently, the cells were treated with Poly IC at concentrations of 2.5, 10, and 25 µg/mL for 22 h to induce the expression of the interferon-induced GTP-binding protein Mx, serving as a proxy for IFN (type-I and type-III) expression ([22], and references therein). Following this incubation, adherent cells were washed with prewarmed HBSS; lysed using 60 µL of M-Per mammalian protein extraction reagent (Pierce; Thermo Fisher Scientific) containing a complete protease inhibitor (Roche Diagnostics, Rotkreuz, Switzerland) and stored at −20 °C for subsequent Western blot analysis. Western blot for the expression of the Mx protein with β-actin as loading control per lane was performed as described by de Martin and Schweizer [57].

In the experimental design, where E^rns^ was applied to the apical side and Poly IC to the basolateral side (or vice versa), the incubation steps were followed as outlined above. This method ensured that E^rns^ was always incubated before the introduction of Poly IC, allowing the protein to enter the cells prior to the initiation of IFN stimulation.

## 3. Results

### 3.1. Poly IC Induces Mx Protein Expression in bBAEC Cultured at the Air–Liquid Interface (ALI)

The initial step in assessing the impact of E^rns^ in the ALI system was to verify the responsiveness of bovine airway epithelial cells to Poly IC stimulation, a synthetic analogue of dsRNA that serves as an agonist for Toll-like receptor 3 (TLR3) and retinoic acid-inducible gene I (RIG-I)-like receptors (RLRs). For this purpose, we used the expression of the Mx protein as a marker for the induction of the IFN pathway.

**Figure 1:**
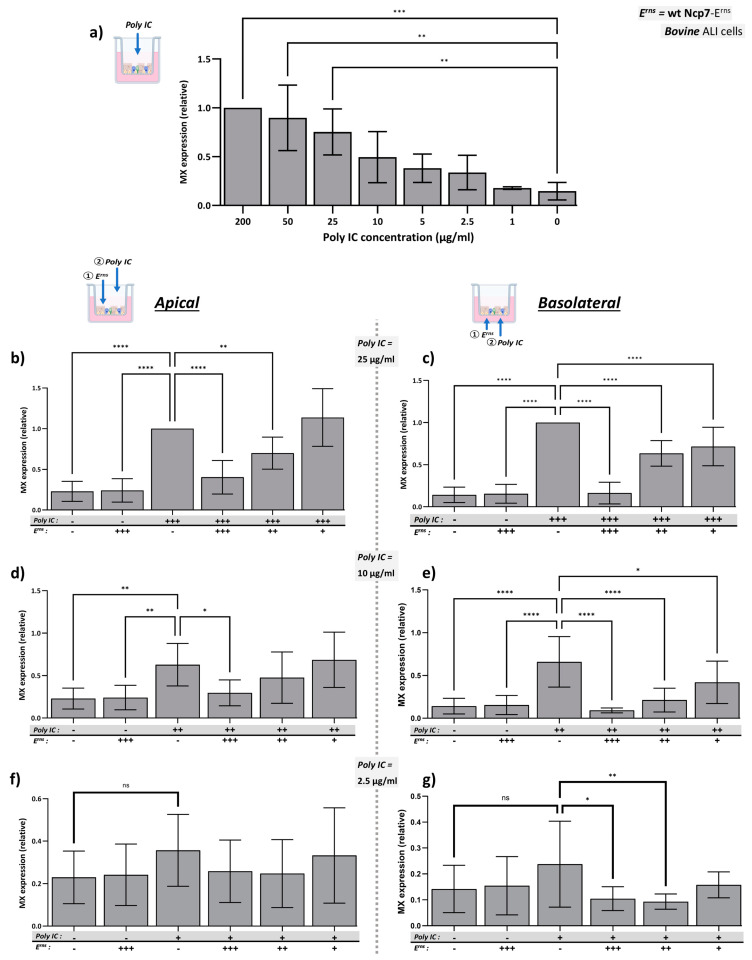
Inhibition of dsRNA-induced Mx expression by E^rns^ in bBAEC cultured in an ALI system. (**a**) Poly IC was applied to the apical side of well-differentiated bBAEC cultures. After 20 h of incubation, cytosolic extracts were assayed for Mx expression by Western blot using β-actin as loading control. The relative expression of Mx at the highest concentration of Poly IC was set to 1 (100%). (**b**–**g**) Strep-tag purified wild-type E^rns^ from the BVDV strain Ncp7 was pre-incubated for 90 min on bBAEC cultures prior to the addition of Poly IC, both to the apical (**b**,**d**,**f**) and basolateral sides (**c**,**e**,**g**). The signal for Poly IC-induced Mx expression was quantified with the one in the absence of E^rns^ present on each gel being set to 1 (100%), and the means of each column was compared to this reference value (mean ± SD, n = 8–9 with cells from 3 different donors). Concentrations indicated in the figure: (+++): 25 μg/mL Poly IC or 25 ng/mL E^rns^; (++): 10 μg/mL Poly IC or 10 ng/mL E^rns^; (+): 2.5 μg/mL Poly IC or 2.5 ng/mL E^rns^; (-): no Poly IC or E^rns^. Ordinary one-way analysis of variance (ANOVA) for multiple comparisons was performed using GraphPad Prism 10.4.0 software with significant differences indicated as follows: **** (*p* < 0.0001), *** (*p* < 0.001), ** (*p* < 0.01), * (*p* < 0.1), or ns (not significant). For clarity of the figure, most of the non-significant comparisons to the reference columns were omitted.

Bovine airway epithelial cells (bBAEC) exhibited a dose-dependent production of Mx protein upon apical stimulation with Poly IC (Figure 1a). The Mx protein production induced by the highest Poly IC concentration applied (200 µg/mL) was used as reference for normalization throughout the results. A clear dose dependence was observed between 2.5 and 25 or 50 µg/mL of Poly IC. Higher concentrations appeared to saturate the level of Mx induction, whereas lower concentration failed to clearly activate the IFN system. Consequently, these three concentrations (2.5, 10, 25 µg/mL of Poly IC) were selected for subsequent experiments.

Notably, the expression of the Mx protein was also regularly detectable in untreated cells, indicating that bovine airway epithelial cells possess a basal level of interferon-stimulated genes (ISGs). This contrasts with previous results using various cell lines, e.g., BT or MDBK cells [29]. Thus, there might be constitutive IFN expression in bBAEC or, alternatively, the ALI culture induces some stress on the cells leading to the activation of the IFN pathways.

### 3.2. E^rns^ Inhibits Poly IC-Induced Expression of Mx in ALI-Cultured bBAEC

The expression of the Mx protein induced by the apical (Figure 1b,d,f) and basolateral (Figure 1c,e,g) addition of Poly IC at 2.5 to 25 µg/mL to the bBAEC was dose-dependently inhibited by E^rns^ of the BVDV strain Ncp7 added to the same side as the dsRNA. The inhibition was more pronounced upon addition to the basolateral side, which might be based on the prolonged incubation of E^rns^ in the medium compared to the apical side. Due to considerable variability of the Poly IC stimulation at low concentrations (Figure 1f,g), the results were only significant in a few cases.

**Figure 2:**
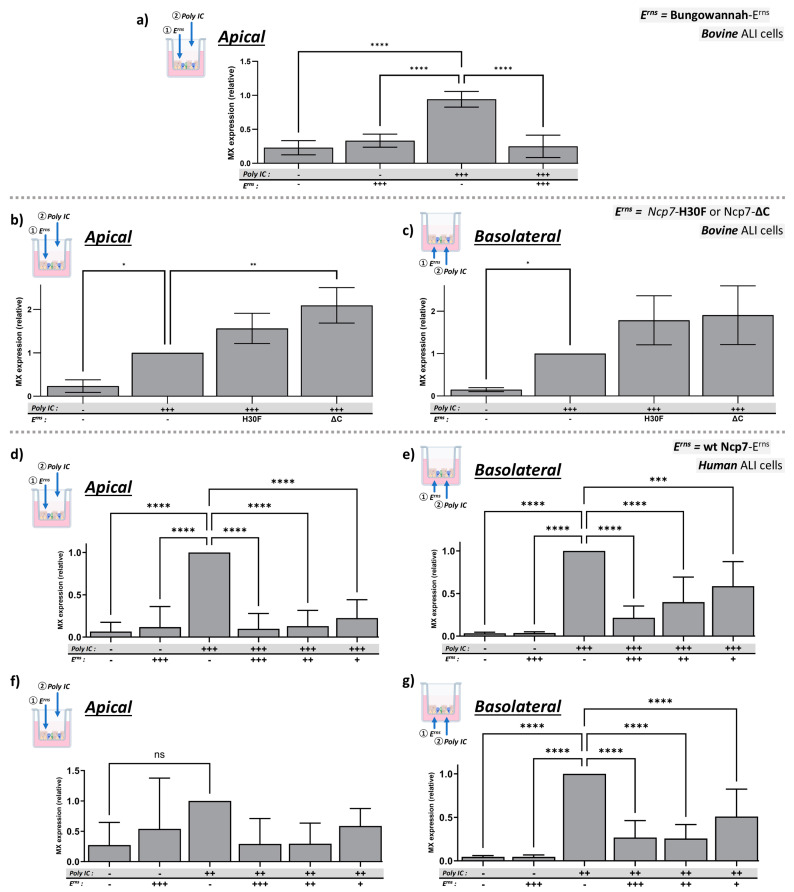
Inhibition of dsRNA-induced Mx expression by wt- or mutant- E^rns^ in bBAEC and hBAEC cultured in an ALI system. (**a**) Strep-tag purified wild-type E^rns^ from the porcine pestivirus Bungo wannah virus (25 ng/mL) was pre-incubated or not for 90 min on the apical side of bBAEC cultures prior to the addition of Poly IC (25 μg/mL) at the same side for another 20 h. (**b**,**c**) RNase-inactive E^rns^ (H30F) or mutant E^rns^ lacking the C-terminal amphipathic helix (ΔC) from the BVDV strain Ncp7 was pre-incubated on the apical (**b**) or basolateral (**c**) side of bBAEC as described in the methods section prior to the addition of Poly IC at 25 μg/mL. (**d**–**g**) Strep-tag purified wild-type E^rns^ from the BVDV strain Ncp7 was pre-incubated for 90 min at the indicated concentrations on human hBAEC cultures prior to the addition of Poly IC at 10 (**f,g**) or 25 μg/mL (**d**,**e**), both at the apical (**d**,**f**) or basolateral side (**e**,**g**). In all panels, the signal for Poly IC-induced Mx expression in the absence of E^rns^, present on every gel, was set to 100% with the mean of each column being compared to this reference column (mean ± SD, n = 3 to 8 with cells from 3 different donors). Ordinary one-way analysis of variance (ANOVA) for multiple comparisons was performed using GraphPad Prism 10.4.0 software with significant differences indicated as follows: **** (*p* < 0.0001), *** (*p* < 0.001), ** (*p* < 0.01), * (*p* < 0.1), or ns (not significant). For clarity of the figures, most of the non-significant comparisons to the reference columns were omitted. Concentrations indicated in the figure: (+++): 25 μg/mL Poly IC or 25 ng/mL E^rns^; (++): 10 μg/mL Poly IC or 10 ng/mL E^rns^; (+): 2.5 ng/mL E^rns^; (-): no Poly IC or E^rns^.

Previously, we showed that E^rns^ proteins from most pestivirus species act as IFN antagonist in cultured bovine cell lines [57]. To confirm and extend these observations, we investigated whether E^rns^ of Bungowannah virus, a pestivirus isolated from pigs in Australia, is also able to inhibit dsRNA-induced IFN expression on bBAEC cultured in an ALI system. Similarly to BVDV-E^rns^, the E^rns^ protein from Bungowannah virus added apically to the ALI cultures completely inhibited Mx expression induced by Poly IC added to the same side (Figure 2a).

### 3.3. Full-Length, RNase-Active E^rns^ Is Required to Inhibit Poly IC-Induced IFN Response in bBAEC

The C-terminal region of E^rns^ contains a positively charged domain within an amphipathic helix that allows its interaction with cell-surface glycosaminoglycans (GAGs). In addition to this GAG-binding site, the RNase activity of E^rns^ was also shown to be required for its IFN antagonistic activity [30,41] in cultured cell lines. Here, we show that the RNase-inactive mutant H30F, and a mutant lacking the C-terminal amphipathic helix were unable to block Poly IC-induced Mx expression also in bBAEC when added at the apical or basolateral side of the ALI cultures (Figure 2b,c). Notably, both inactive mutants appear to even enhance dsRNA-induced Mx expression, an unspecific effect that we already observed previously [28,30] upon the addition of concentrated solutions of inactive E^rns^ preparations.

### 3.4. E^rns^ Inhibits Poly IC-Induced Mx Protein Expression in Human Bronchial Airway Epithelial (hBAEC) Cultured in an ALI System

GAG-binding per se is not species specific, and accordingly, we previously showed that E^rns^ is able to inhibit dsRNA-induced activation of the IFN system in bovine as well as in ovine, caprine, canine, and human cell lines [30]. Here we show that BVDV-E^rns^ is similarly able to dose-dependently inhibit Mx expression induced by 10 and 25 µg/mL Poly IC added at the apical or basolateral side of human airway cells cultures in an ALI system (Figure 2d–g). The stimulation by the reduced amount of Poly IC (Figure 2f) added to the apical side was rather low, probably based on the short incubation time with the dsRNA (compare Figure 1), yielding in most cases to non-significant inhibition by E^rns^.

### 3.5. E^rns^ Reduces the Expression of Mx Protein in bBAEC After Stimulation by Poly IC on the Opposite Side

**Figure 3:**
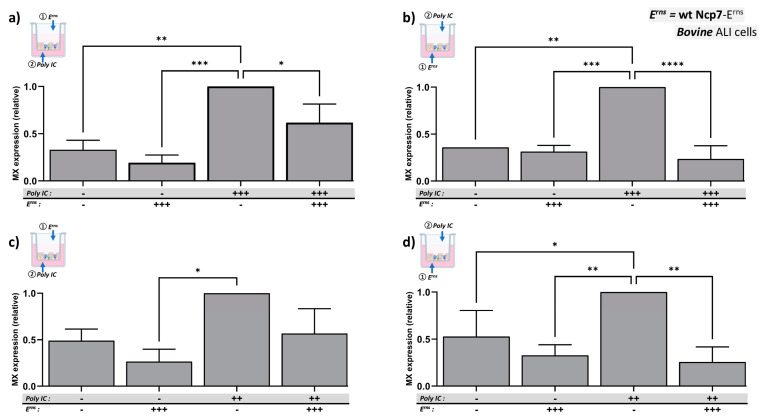
Inhibition of dsRNA induced Mx expression by E^rns^ added to opposite sides of bBAEC cultures. Strep-tag purified wild-type E^rns^ from BVDV strain Ncp7 at 25 ng/mL (+++) was pre-incubated on the apical side of the bBAEC cultures for 90 min followed by its removal and further incubation with the apical side exposed to air for a total of 24 h (**a**,**c**). On the basolateral side (**b**,**d**), this E^rns^ was incubated with the bBAEC cultures for the complete incubation time of 24 h. Thereafter, Poly IC was added at 25 μg/mL (+++) (**a**,**b**) or 10 μg/mL (++) (**c**,**d**) to the opposite side compared to E^rns^. On the basolateral side, the dsRNA was incubated for 20 h, whereas on the apical side, Poly IC was removed after 2 h followed by 18 h of incubation with the apical side exposed to air. Thereafter, cytosolic extracts were assayed for Mx expression by Western blotting using β-actin as the loading control. The signal for Poly IC-induced Mx expression in the absence of E^rns^ present on each gel was set to 1 (100%), and the mean of each column was compared to this reference column (mean ± SD, n = 3 to 6 with cells from 3 different donors). Ordinary one-way analysis of variance (ANOVA) for multiple comparisons was performed using GraphPad Prism 10.4.0 software with significant differences indicated as follows: **** (*p* < 0.0001), *** (*p* < 0.001), ** (*p* < 0.01), * (*p* < 0.1). For clarity of the figure, the non-significant comparisons to the reference columns were omitted.

To investigate whether E^rns^ needs to be added to the same side as the dsRNA in order to inhibit the activation of the IFN system, we pre-incubated E^rns^ basolaterally for 24 h and then stimulated the bBAEC with Poly IC for 2 h on the opposite apical side (Figure 3b,d) and vice versa (Figure 3a,c). However, the ALI cultures of bBAEC did not tolerate the presence of liquid in the apical space instead of the usual air exposure for longer time, and, thus, we pre-incubated E^rns^ solely for 3 h on the apical side, the maximum time tole ra ted by the cultures, before exposing this side to air again for another 21 h.

We observed that for both concentrations of Poly IC, there was indeed an inhibition of the Mx protein expression in the presence of E^rns^ pre-incubated on the opposite side compared to the side of Poly IC addition. However, the effect appeared to be more pronounced when E^rns^ was pre-incubated on the basolateral side, most probably based on its much longer pre-incubation time and, thus, more of the E^rns^ protein might have entered the cells. However, E^rns^ could not be incubated for a longer time on the apical side without damaging the integrity of the cell layer.

### 3.6. Basolateral Release of Pestiviral RNA upon Infection of bBAEC by Different Strains of BVDV

In order to investigate whether infection of bBAEC cultures by BVDV produces sufficient levels of E^rns^ to inhibit dsRNA-induced activation of the IFN system, we first had to characterize the infection of the pseudostratified bovine airway cells cultured in the ALI system [44]. Therefore, we infected bBAEC cultures with various noncytopathogenic strains of BVDV, i.e., Ncp7, Pe515, Suwa, and NADL, and with Bungowannah pestivirus. After 72 h, immunofluorescence staining for IDV was readily observed (not shown) as reported [44], whereas even with several attempts, we could never observe a specific staining for any pestiviral antigen after 3 days of infection (not shown).

To test for the possibility that infection and replication of BVDV in these cultures is delayed compared to infection with IDV, we prolonged the incubation after infection for up to 5 days post-infection (dpi) and analyzed the synthesis and release of viral RNA by real-time RT-PCR for increased sensitivity.

**Figure 4:**
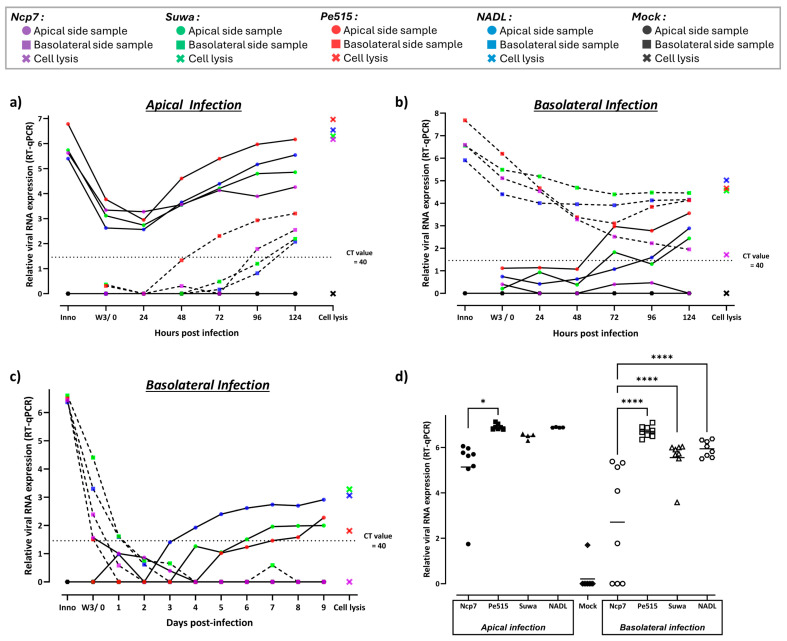
Detection of viral RNA of different BVDV strains after infection of bBAEC cultured in an ALI system. (**a**–**c**) Two different bovine donors were infected with four different BVDV strains (Ncp7, Pe515, Suwa, NADL) apically and basolaterally for 5 days (**a**,**b**) or basolaterally for 9 days (**c**) (n = 3). Infection was monitored with a sample obtained every 24 h on each side (apical, continuous line; basolateral, dashed line). (**d**) Cells from an additional, freshly isolated bovine donor were included. The bBAEC from the three different donors were infected with the same BVDV strains apically and basolaterally as before. After 10 days of incubation (without intermittent sampling in order not to stress the pseudo-stratified layer), cells were lysed and analyzed (n = 4–9). Total RNA from all samples (**a**–**d**) was extracted, purified, and analyzed by RT-qPCR for the quantification of pestiviral RNA. The relative amount of viral RNA based on the Ct value was obtained as described in the Materials and Methods section. Statistical analysis was as described in Figure 3.

After apical infection of the bBAEC cultures, the release of viral RNA was observed starting at 48 to 72 h dpi (Figure 4a). However, the release was much more pronounced from the apical side (continuous line) than from the basolateral side (dashed line), with the largest amount of RNA detected on both sides originating from the Pe515 strain. Upon basolateral infection of the bBAEC cultures, the release of viral RNA from the apical side started only at 72 h dpi and occurred to a much smaller extent than after apical infection (continuous line), except for the strain Ncp7 that failed to release viral RNA. By contrast, there was only a steady decrease in the amount of viral RNA detected on the basolateral side (dashed line) after basolateral infection, indicative of a lack of active release of newly synthesized viral RNA (Figure 4b). Lysis of the cells after 5 days followed by analysis of the viral RNA indicated that the cultures could indeed be infected from both sides, but that the infection and replication was much more efficient after apical infection compared to infection from the basolateral side (Figure 4a,b). In accordance with the lack of release of viral RNA from the strain Ncp7 after basolateral infection, the amount of viral RNA in the cell lysate was only marginally above the detection limit (Figure 4b).

To exclude the possibility that the replication and release of viral RNA from the BVDV strain Ncp7 was just slower than for the other strains, we further extended the incubation time to 9 dpi after infection from the basolateral side (Figure 4c). But once again, there was no release of viral RNA for the Ncp7 strain on either side of the bBAEC (Figure 4c), whereas viral RNA of the strains NADL, Suwa, and Pe515 could be observed on the apical side starting at 3 to 6 dpi and leveling off at 6 to 7 dpi. Analysis of the cell lysates at 9 dpi was negative for the strain Ncp7 only, indicating again that this strain was not or only barely able to infect the bBAEC from the basolateral side.

However, at least one repetition of infection of the bBAEC with any pestivirus strain tested failed to release viral genomes and, thus, appeared to be unable to establish infection. Thus, in addition to the virus strain differences, with Ncp7 being largely unable to infect the cells, there appears to be quite a variability by the donor cells in their efficiency of establishing the pseudostratified bovine airway cell layer and their susceptibility to infection, with the majority of negative real-time RT-qPCR results originating from a single donor.

To further validate the donor variability, we prepared cells from a new, additional donor and infected them from the apical and basolateral side as above. However, we exclusively analyzed the cell lysates after 10 dpi in order to avoid any stress on the ALI cultures by the daily sampling, especially from the apical side (Figure 4d). Apparently, bBAEC could be infected from both sides, apical and basolateral, readily supporting the replication of the strains Pe515, Suwa, and NADL. But again, the strain Ncp7 was not able to infect the cells in all cases, and after productive infection, this strain replicated to levels significantly lower than the other three strains.

### 3.7. Apical Release of Infectious Pestivirus Particles upon Infection of bBAEC

**Figure 5:**
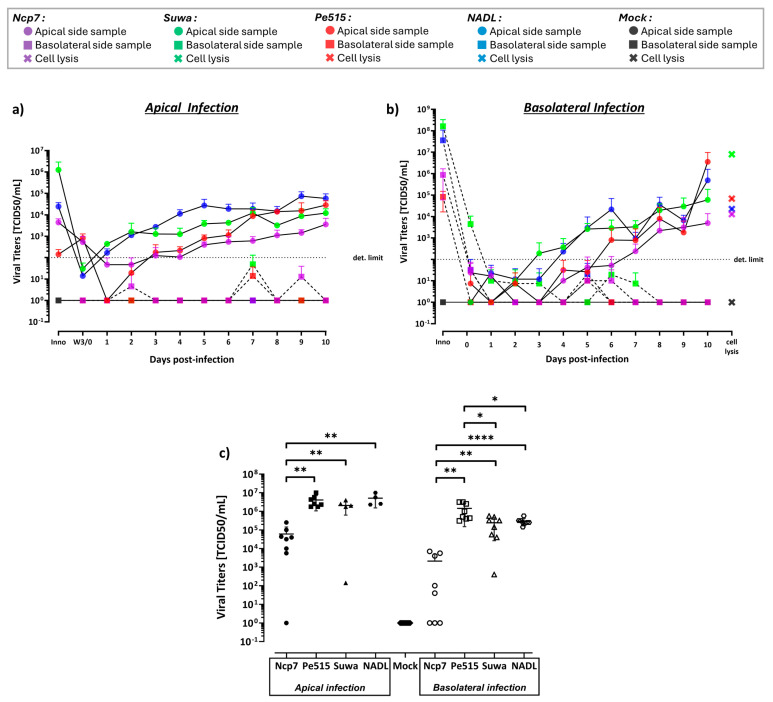
Detection of infectious BVD viruses after infection of bBAEC cultured in an ALI system. (**a**,**b**) Two bovine donors were infected with four different BVDV strains (Ncp7, Pe515, Suwa, NADL) apically (**a**) and basolaterally (**b**) for 10 days (n = 6). Infection was monitored with a sample obtained every 24 h on each side (apical, continuous line; basolateral, dashed line). (**c**) Cells from an additional, freshly isolated bovine donor were included. The bBAEC from the three different donors were infected with the same BVDV strains apically and basolaterally. After 10 days of incubation (without intermittent sampling), cells were lysed and analyzed (n = 4–8). The virus titer of all samples was determined by titration on BT cells as described in the Materials and Methods section. Ordinary one-way analysis of variance (ANOVA) for multiple comparisons was performed using GraphPad Prism 10.4.0 software with significant differences indicated as follows: **** (*p* < 0.0001), ** (*p* < 0.01), * (*p* < 0.1). For clarity of (**c**), the significant differences of the virus titers to uninfected cells (Mock) (*p* < 0.001 for Ncp7; *p* < 0.0001 for Pe515, NADL, and Suwa) were omitted.

In addition to the analysis with real-time RT-PCR, which measures only viral RNA independent on its infectivity, we analyzed the release of infectious virus particles on the apical and basolateral side after infection from either side by virus titration. Regardless of the initial side of infection and the virus strain used, i.e., Ncp7, Suwa, Pe515, or NADL, newly assembled infectious viruses were only detected in samples from the apical chamber (Figure 5a,b). No infectious viruses were detected in almost all samples that were obtained from the basolateral chamber except a low-virus titer detected late after infection (7 and 9 dpi) by the strains Pe515 and Suwa after apical infection (Figure 5a). Measurements of TEER at day 0 and day 9–10 dpi indicated no significant differences between infected and non-infected bBAEC cultures throughout the experiments, suggesting that the barrier function separating the apical and basolateral side remained unaffected by pestivirus infection.

In order to quantify productive infection of the bovine air–liquid interface cultures without disturbing the cell layer by frequent washings, we analyzed only the cell lysates after infection for 10 days with the different BVDV strains (Figure 5c). Whether upon basolateral or apical infection, a significant number of infectious virus particles were found within the cells for all BVDV strains compared to the negative control. The previous observation of the limited ability of Ncp7 to replicate after basolateral infection, analyzed by real-time RT-qPCR (Figure 4), could be confirmed by the significant decrease in detectable infectious virus particles in cells compared to other BVDV strains (Figure 5c). Indeed, it was not possible to detect Ncp7 infectious virus particles intracellularly in a number of experimental replicates even 10 days after infection.

### 3.8. Infection of bBAEC by Different Pestiviruses Analysed by Immunofluorescence Microscopy

**Figure 6:**
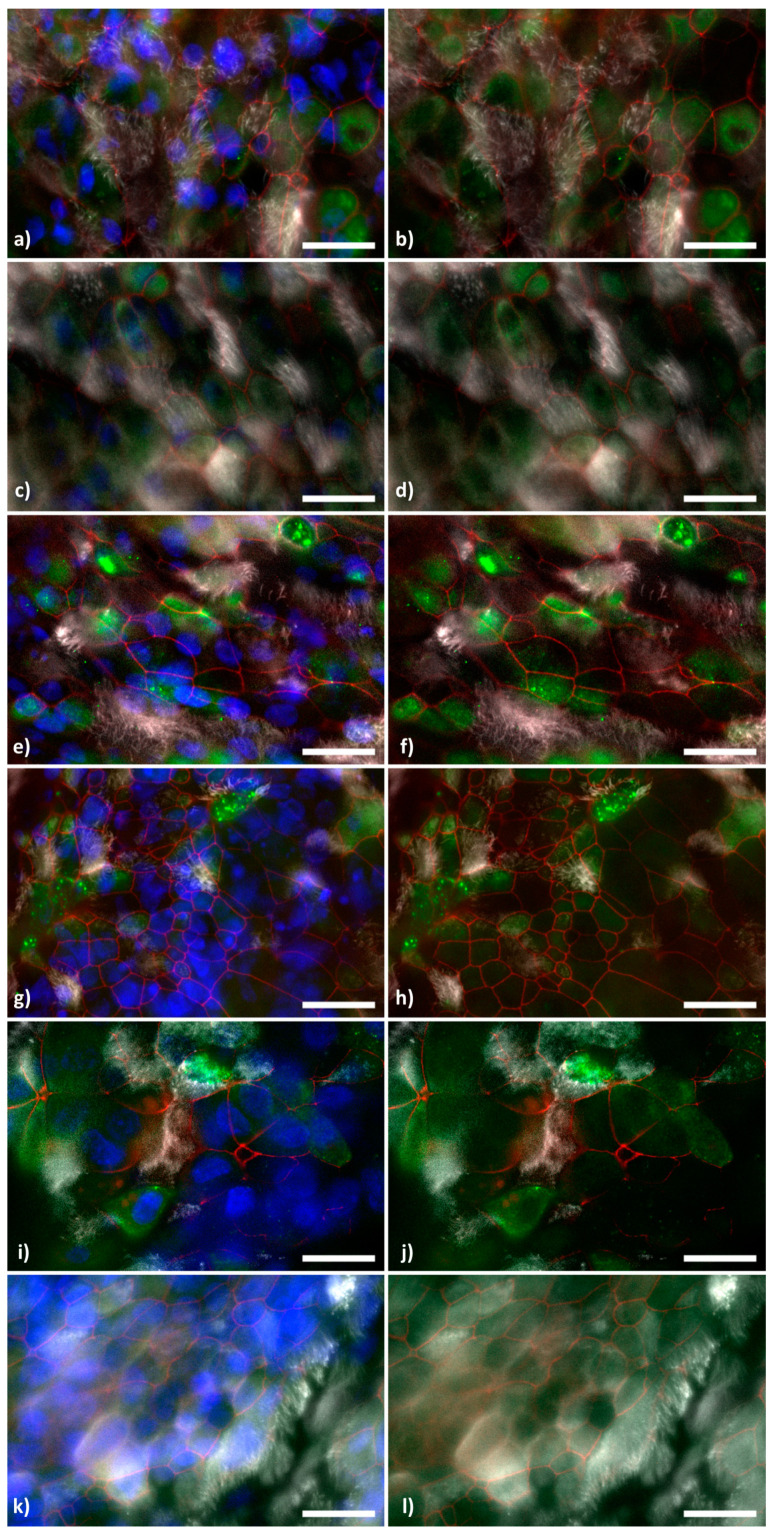
Detection of BVDV after infection of bBAEC cultured in an ALI system by immunofluorescence microscopy. Well-differentiated bBAEC were infected with the BVDV strains Ncp7 (**a**,**b**), Pe515 (**c**,**d**), Suwa (**e**,**f**), or NADL (**g**,**h**), with Bungowannah pestivirus (**i**,**j**) or left uninfected (**k,l**) for 10 days (n = 3). Thereafter, the cells were stained as described for the nuclei (blue), tight junctions (red), cilia (white), and dsRNA (green). Scale bar = 20 μm. For better visibility of the virus-infected cells (green channel), each picture is presented with all color channels (**a**,**c**,**e**,**g**,**i**,**k**) or without the nuclei staining (blue channel) (**b**,**d**,**f**,**h**,**j**,**l**).

To confirm the infection of the cells cultured in an ALI system, the cells were infected apically and basolaterally with different pestivirus strains, i.e., the non-cytopathogenic BVDV-1 strains Ncp7, Pe515, Suwa, and NADL, (Figure 6a–h) and Bungowannah pestivirus (Figure 6i,j), for 10 days prior to fixation and analysis by immunofluorescence microscopy. Due to the limited availability of ALI cell cultures, and in light of the fact that in the previous experiments, we did not observe a substantial difference between the infection of the cultures from either side (Figure 4 and Figure 5), we infected the cultures from the apical side with the strains Pe515, Suwa, and NADL, whereas the Ncp7 and Bungowannah pestiviruses were applied from the basolateral side. Using a sensitive, commercially available combination of primary antibodies to the structural protein E2 and the non-structural protein NS2-3 (not shown), or an antibody to dsRNA (Figure 6), we were able to detect positive signals for all the viruses tested in the bovine cells cultured in the ALI system. There was no difference in staining using antibodies to viral antigens or to dsRNA, and we found ciliated and non-ciliated cells to be infected. As only a small number of cells were infected, a clear, specific cell tropism, however, could not be assigned by these experiments.

## 4. Discussion

BVD viruses are successfully transmitted between their host animals by transiently (acutely) and persistently infected animals, with the generation of PI animals guaranteeing the long-term maintenance of the virus in the host population. For the latter, the expression of the two IFN antagonists, i.e., N^pro^ and E^rns^, is essential to successfully infect the fetus and establish immunotolerance required for persistence ([13], and references therein). In both types of infection, infectious virus is shed from all secretions, such as saliva, tears, semen, milk, and possibly feces. Oronasal transmission between infected and naive animals is the principal way of horizontal infection. E^rns^, in addition to its role as envelope glycoprotein, is released in a soluble form into the same secretions, which is also used diagnostically as a surrogate for viral particles [58]. As BVDV is regularly associated with respiratory symptoms summarized as bovine respiratory disease complex (BRDC), it might be well envisaged that E^rns^ plays a role in suppressing the innate immune system in the respiratory tract. Thus, we aimed to investigate the inhibition by E^rns^ of the dsRNA-induced activation of the host’s IFN response in an in vitro model mimicking the natural situation of the bronchial epithelium by culturing the cells as a pseudostratified, well-differentiated layer cultured in an air–liquid interface (ALI) system [46,59].

Here, we show that BVDV-E^rns^ dose-dependently inhibits Poly IC-induced Mx expression (Figure 1), a well-established surrogate marker for the presence of type-I and type-III IFN. It did not depend on the side of application, i.e., E^rns^ inhibited the IFN system upon apical and basolateral addition. In addition to BVDV, E^rns^ of the only distantly related Bungowannah pestivirus similarly blocked dsRNA-induced Mx expression (Figure 2a), confirming that most if not all the pestiviral RNases act as IFN antagonist [57]. As reported previously [27,30,41,56], the RNase-inactive mutant (E^rns^-H30F) and a mutant lacking the C-terminal GAG-binding site (E^rns^-ΔC) were unable to block innate immune activation (Figure 2b). The latter is corroborated by the fact that binding and cell entry of E^rns^ does depend rather on unspecific binding by its C-terminally located, positively charged amphipathic helix than on a species-specific receptor, as BVDV-E^rns^ was similarly active in human airway epithelial cells cultured at ALI (Figure 2d–g), as shown previously with human, ovine, caprine, or canine cell lines [30,41,60]. On both, hBAEC and bBAEC, a certain ratio of the amount of Poly IC and E^rns^ had to be maintained (e.g., a low amount of E^rns^ was unable to block the highest concentration of Poly IC), and the efficacy of E^rns^ was more pronounced when applied basolaterally (Figure 1 and Figure 2). The latter is probably not based on a higher affinity of E^rns^ to the basolateral side but rather to the longer incubation time, as on the apical (air-exposed) side, medium with E^rns^ or Poly IC could be added for a maximum of three hours before deterioration of the cell layer occurred. Notably, E^rns^ was similarly inhibiting dsRNA-induced Mx expression when added on the opposite side of the cell layer (Figure 3), though less efficiently. By definition, a ‘pseudostratified’ epithelium consists of only one layer, with all cells adhering to the basal membrane. Whether this is the absolute case after differentiation cannot be proven (e.g., compare [59]). Therefore, we cannot state with certainty that each cell is accessible by E^rns^ from both sides, or whether transmission of the E^rns^ protein from cell to cell might occur. The latter was not reported to date for the E^rns^ protein, but evidence for cell-to-cell transmission of BVDV in cell cultures has been provided [61]. Nonetheless, this indicates that E^rns^ acts intracellularly in an endolysosomal compartment (for review, see [25] and references therein). This supports previous observations that complexed dsRNA protected from RNase degradation in suspension in vitro can still be inhibited by intracellular E^rns^ [60] and that the C-terminal domain appears to be sufficient to determine the intracellular location, as GFP fused to the C-terminal amino acids of E^rns^ can be found at the same location as wt E^rns^ [41].

In order to test whether infection of bBAEC with BVDV produced a sufficient amount of E^rns^ to block dsRNA-induced IFN expression, we infected the epithelial cell cultures at ALI prior to the addition of Poly IC. However, infection of the bBAEC for 7 days did not yield any reduction in Poly IC-induced Mx expression, independent of whether the cells were infected apically or basolaterally (not shown). Therefore, we characterized the infection of the bBAEC by analyzing the presence of viral RNA by RT-qPCR, of infectious virus by titration, and of infected cells by immunofluorescence microscopy. Only after prolonged incubation, we were able to detect viral RNA and infectious virus particles in samples obtained from both sides of the polarized cell layer (Figure 4 and Figure 5). Thus, the bBAEC cultured in an ALI system could be infected from both sides, with the infection being somewhat more efficient from the apical side. This is in accordance with [40], where apical infection yielded slightly higher titers in the apical compartment, whereas infection from the basolateral compartment appeared to spread better between the cells in the non-differentiated, polarized cell layer. However, in our differentiated bBAEC culture at the air–liquid interface, infectious BVDV was only actively released from the apical side, and not or only barely from the basolateral side, as reported for non-differentiated, polarized primary bovine airway epithelial cells [40]. By contrast, viral RNA could be observed on the apical side after apical infection, whereas only input viral RNA could be detected on the apical side for several days upon basolateral infection (Figure 4). Whether this viral RNA in the absence of infectious virus originates from defective particles or was released from damaged or dying cells is not known. In addition to the variability by the donors of the bBAEC, we observed quite a heterogeneity between the different BVDV strains, with Ncp7 being the least and Pe515 the most efficient strain to infect the cultures. This could be confirmed by infection of the bBAEC for ten days without intermittent sampling to preserve the cell layer followed by analysis of the cell lysates. The amount of viral RNA (Figure 4d) and infections virus (Figure 5c) detected in the cell lysates was significantly lower for the strain Ncp7, with some replicates even being negative. The cause of these strain differences is currently unknown, but for the strain NCP7, it might be related to a strong adaptation to various cell lines, with an unknown passage history over several decades [62]. Immunofluorescence microscopy after 10 days of infection confirmed successful infection of the differentiated bBAEC (Figure 6). The fact that only a limited number of cells are infected despite a considerable titer of infectious virus observed in the cell lysates indicates that BVDV replicates rather efficiently in the cells targeted. The infection was not different using Bungowannah pestivirus (Figure 6i,j), despite this species being reported to have a rather broad cell and species tropism [63]. However, the precise characterization of the cell tropism was beyond the scope of this study, and this aspect was not investigated in more detail.

In this study, we provide evidence that the viral RNase E^rns^ is able to inhibit the activation of the host’s IFN response induced by dsRNA when added on either side (apical and basolateral) of differentiated airway epithelium cultured in an ALI system1 [46,59]. E^rns^’ activity as IFN antagonist requires its RNase activity and the GAG-binding site in the C-terminal domain. The latter appears to be sufficient to guide E^rns^ into an intracellular, endolysosomal compartment, i.e., to the same location as Poly IC, as both can even be added to opposite sides of the polarized cell layer. There, E^rns^ degrades viral PAMPs, i.e., highly structured ssRNA and dsRNA, prior to their activation of TLR-3 and TLR-7/8 [25]. This appears to be the case for most if not all E^rns^ proteins from all pestiviruses, as indicated in this study by the viral RNase of BVDV and of Bungowannah virus, and as shown previously in cell lines using a large variety of pestivirus species [57]. Notably, the specific type of glycosylation of E^rns^ appears to be of minor importance for its activity as IFN antagonist, as E^rns^ produced in insect, bovine, or human cells exert similar activity [27,29,56]. The importance of E^rns^ as IFN antagonist for pestiviruses is further supported by the fact that this viral RNase is present in all viruses within the genus *Pestivirus*, even in the Phocoena pestivirus that is the only currently known pestivirus that lacks the second IFN antagonist, N^pro^ [64]. As E^rns^ can be found on both sides of the respiratory epithelium, e.g., in the blood and saliva, it can be well envisaged that it substantially contributes to the immunosuppressive effect observed after BVDV infections [33], especially in PI animals, where viral antigen was indeed detected in the bronchial epithelium [65], and where we found around 50 ng/mL free E^rns^ protein in the blood [28].

The infection of the ALI cells could be achieved from the apical and basolateral side of the cell layer; however, infectious virus was only released from the apical side. Thus, together with the rather inefficient infection of this cell culture model requiring several days for successful infection, the precise way of infection with BVDV by the oronasal route remains to be established. This also shows the limitation of this cell culture model, despite it mimicking many aspects of the respiratory tract, such as the presence of ciliated and other cells, mucus production, and innate immune activation [46], it lacks the presence of immune cells, e.g., macrophages or dendritic cells, that might be involved in oronasal infection with BVDV [40,66]. Nevertheless, infectious virus particles are readily released from the apical side, which might explain the efficient respiratory transmission from infected animals. Nevertheless, primary bronchial epithelial cells cultured at ALI will be an attractive model that can be further expanded to investigate respiratory infections and co-infections [66].

## Data Availability

The original contributions presented in the study are included in the article, further inquiries can be directed to the corresponding author. The raw data supporting the conclusions of this article will be made available by the authors on request.
